# Deformation understanding in the Upper Paleozoic of Ventana Ranges at Southwest Gondwana Boundary

**DOI:** 10.1038/s41598-021-99087-1

**Published:** 2021-10-21

**Authors:** Guadalupe Arzadún, Renata Nela Tomezzoli, Natalia Fortunatti, Nora Noemi Cesaretti, María Belén Febbo, Juan Martin Calvagno

**Affiliations:** 1 Consejo Nacional de Investigaciones Científicas y Técnicas (CONICET), Laboratorio de Termocronología (LaTe Andes), Las Moreras 310, A4401XBA Vaqueros, Salta Argentina; 2 Consejo Nacional de Investigaciones Científicas y Técnicas (CONICET), Laboratorio de Paleomagnetismo “Daniel A. Valencio”, Instituto de Geociencias Básicas, Aplicadas y Ambientales de Buenos Aires (IGEBA), Buenos Aires, Argentina; 3 Departamento de Ciencias Geológicas, Facultad de Ciencias Exactas y Naturales (FCEyN), Universidad de Buenos Aires (UBA), Pabellón II (1428), Buenos Aires, Argentina; 4 Departamento de Geología, Universidad Nacional del Sur, Av. Alem 1253, Piso 1, Oficina 108, 8000 Bahía Blanca, Argentina; 5 Centro de Geología Aplicada, Agua y Medio Ambiente (CGAMA)-(CIC), Comisión de Investigaciones Científicas, Buenos Aires, Argentina

**Keywords:** Geology, Tectonics

## Abstract

At the east of the Ventana Ranges, Buenos Aires, Argentina, outcrops the Carboniferous-Permian Pillahuincó Group (Sauce Grande, Piedra Azul, Bonete and Tunas Formation). We carried out an Anisotropy of Magnetic Susceptibility (AMS) study on Sauce Grande, Piedra Azul and Bonete Formation that displays ellipsoids with constant K_max_ axes trending NW–SE, parallel to the fold axes. The K_min_ axes are orientated in the NE–SW quadrants, oscillating from horizontal (base of the sequence-western) to vertical (top of the sequence-eastern) positions, showing a change from tectonic to almost sedimentary fabric. This is in concordance with the type and direction of foliation measured in petrographic thin sections which is continuous and penetrative to the base and spaced and less developed to the top. We integrated this study with previous Tunas Formation results (Permian). Similar changes in the AMS pattern (tectonic to sedimentary fabric), as well as other characteristics such as the paleo-environmental and sharp curvature in the apparent polar wander path of Gondwana, marks a new threshold in the evolution of the basin. Those changes along the Pillahuincó deposition indicate two different spasm in the tectonic deformation that according to the ages of the rocks are 300–290 Ma (Sauce Grande to Bonete Formation deposition) and 290–276 Ma (Tunas Formation deposition). This Carboniferous-Permian deformation is locally assigned to the San Rafael (Hercinian) orogenic phase, interpreted as the result of rearrangements of the microplates that collided previously with Gondwana, and latitudinal movements of Gondwana toward north and Laurentia toward south to reach the Triassic Pangea.

## Introduction

**Figure 1:**
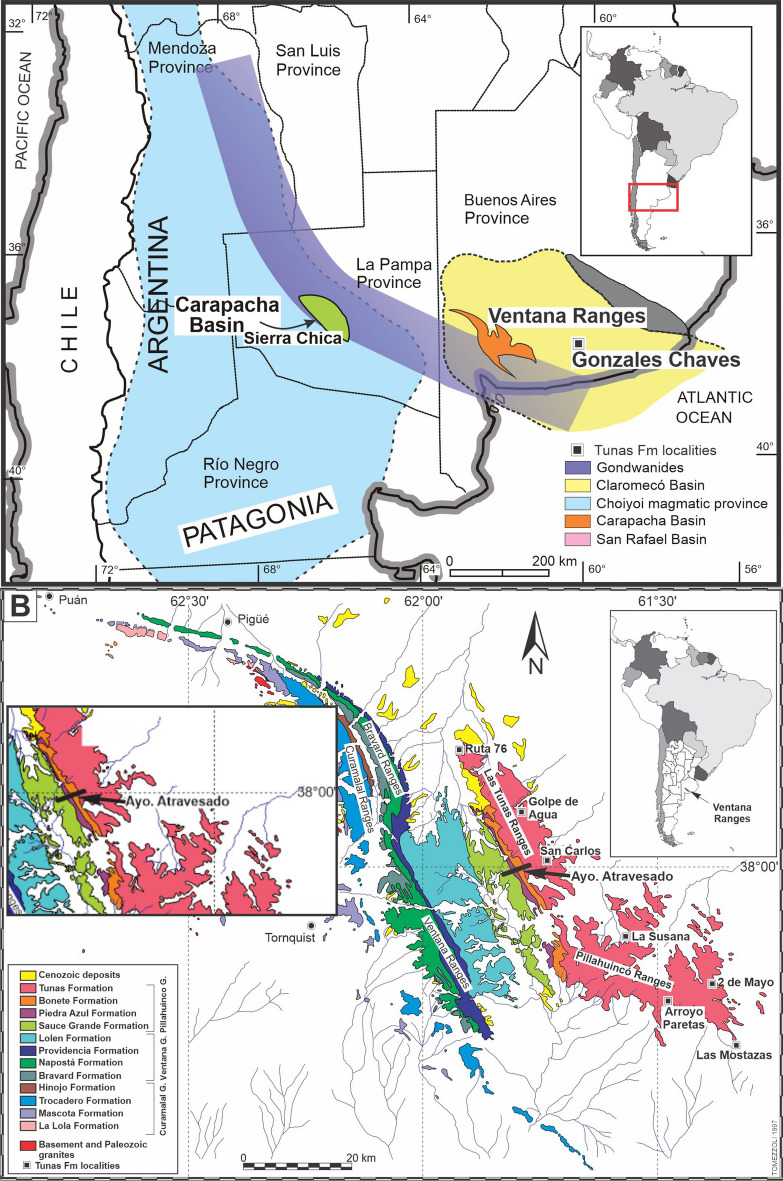
(**A**) Location of the Ventana Ranges thrust and fold belt in the southwest of Buenos Aires province, Argentina and others surroundings geological provinces (Carapacha Basin and Patagonia "terrain"). The study area is the exposed portion of the Claromecó Basin (in yellow) developed in the southwestern margin of the Gondwanides belt (in violet). (**B**) Geologic map of the Ventana Ranges modified from Suero^8^. Location of the Arroyo Atravesado log, where the samples were taken for the AMS studies. Up at the left: enlargement of the work area. The maps were generated and edited with Inkscape (https://inkscape.org/es/).

The Ventana Ranges were part of a larger system interpreted as part of the Hesperides Basin (Pennsylvanian to Lower Triassic), which is in lateral continuity with the Kalahari, Karoo (Africa) and Chaco-Paraná basins (South America), with a depocenter of more than 3,000,000 km^2^^1–3^. They are located in Buenos Aires Province, placed 37°–39° south latitude and 61°–63° west longitude and constitutes an exposed portion of the Claromecó Basin^4–7^ (Fig. 1).

Several authors discuss the timing and intensity of the deformation, and the direction of tectonic stress, in the Ventana Ranges area. Harrington^9^ interpreted the structure as a system formed purely by folding. Rossello and Massabie^10^ suggested a coaxial deformation model and interpreted the structures as the result of a non-rotational pure shear deformation. Other authors proposed a non-coaxial deformation model, with conditions of deformation dominated by simple shear^11–17^. Cobbold et al.^11^ proposed a sinistral model with transpressive motion. Ramos^7^, Chernicoff et al.^18^ and Ramos et al.^19^ remarked that the Ventana Ranges configuration is due to an intercontinental collision between Patagonia and Gondwana. Other authors suggest a system of continental blocks, which moved because of a tectonic event that produced crustal fragmentation through transform faults^16,20–22^. Tomezzoli^23^ suggests that the deformation in the area might be a combination of both processes: collision and subsequent accommodation of the involved plates. Deformation style of the Curamalal, Bravard and Ventana Ranges (Fig. 1), is characterized by a NW–SE asymmetric folding (1C type,^24^. In the eastern sector, Las Tunas and Pillahuincó Ranges (Fig. 1), display a characteristically open folding (syn-kinematic folding,^24^.

Based on paleomagnetic results^25,26^, re-crystallization ages of illite^27^ and growth strata presence^28^, the age of the Ventana Ranges deformation has been assigned to the Permian. Although for some authors, the deformation possibly began during the Devonian–Carboniferous^10,23,29^.

**Figure 2:**
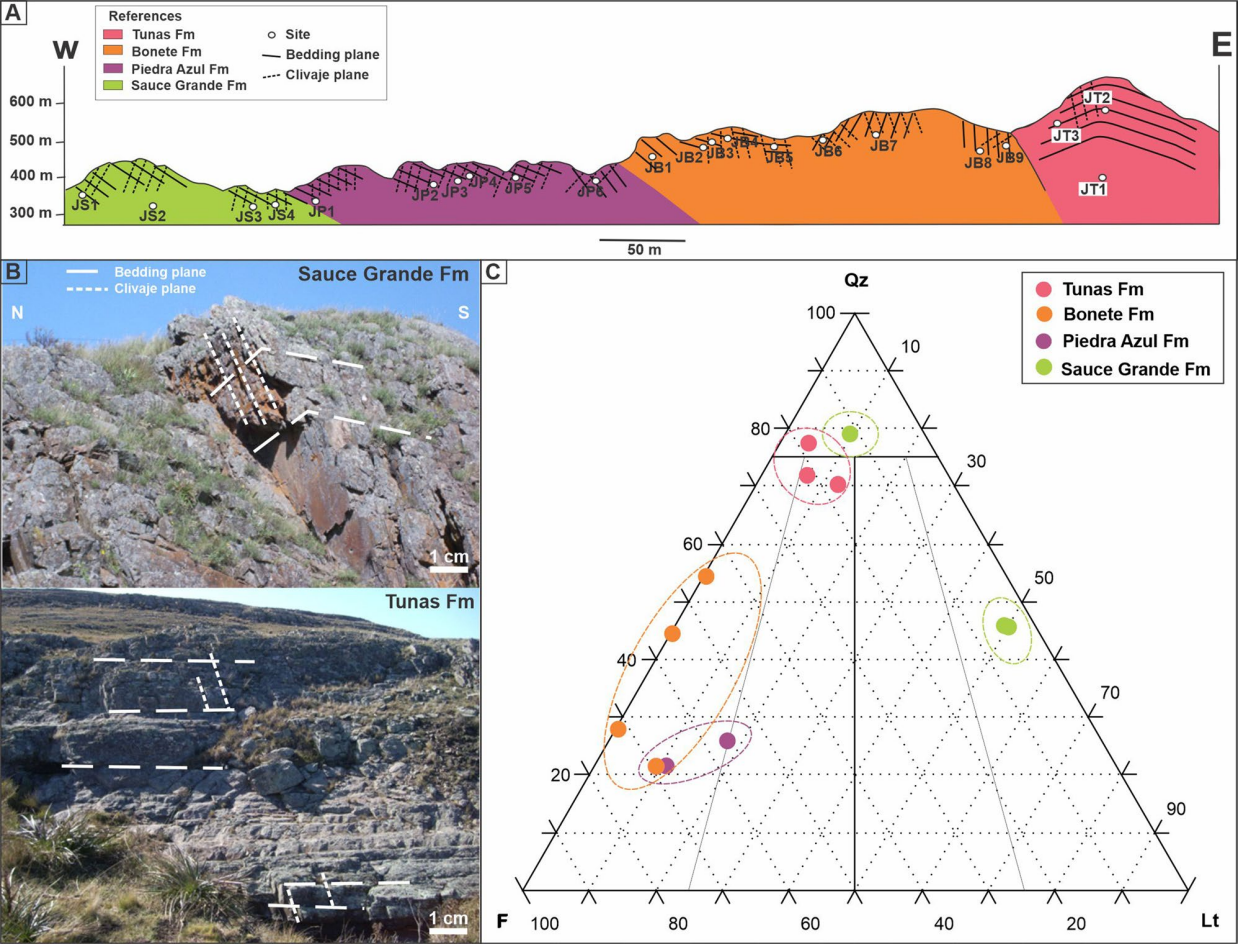
(**A**) Schematic reconstruction of the Arroyo Atravesado section (see Fig. 1), with the different formations of the Pillahuincó Group, its bedding and foliation. *JS* Sauce Grande sites, *JP* Piedra Azul sites, *JB* Bonete sites, *JT* Tunas sites. (**B**) Photographs show the differences of the folding at the base (Sauce Grande Fm, outcrop from the Road 76, near Ruta 76 locality) and at the top (Tunas Fm, outcrop from Golpe de Agua locality). (**C**) Petrographic plot of the analysed samples. *Qz* quartz, *F* feldspar, *Lt* total lithic fragments.

In this contribution, petrographic and AMS analysis were applied in samples from the Carboniferous-Permian sequence, belonging to the Pillahuincó Group, in order to get a better comprehension of the timing and intensity of the deformation in the area. In the Arroyo Atravesado section (Fig. 1B), the entire sequence of the Pillahuincó Group outcrops, from Sauce Grande Formation of Carboniferous age at the west, to the base of the Tunas Formation of Permian age, at the east (Fig. 2). The data obtained in this section were compared with previous AMS results obtained in other localities of Tunas Formation itself^30,31^, in the subsurface of the Claromecó basin Febbo et al.^32^ and from the Carapacha Basin^33^, to have a regional assessment of the deformation evolution along the southwestern Gondwana margin. Further results were compared with theoretical models of Saint-Bezar et al.^34^, Parés and van der Pluijm^35^ and Weil and Yonkee^36^.

## Geological setting

The Ventana Ranges comprises a fold and thrust belt with sigmoidal shape and a northeast vergence in present geographical coordinates^17,37,38^. They are composed by rocks from Late Precambrian in the west to Permian successions in the east (Fig. 1). The stratigraphic sequence is divided into three main stratigraphic units: Curamalal, Ventana and Pillahuincó groups, which show important differences in metamorphism degree and style of the deformation between them^9^ (Fig. 1). The older lithologies belong to the Curamalal and Ventana Groups that are situated on the western sector (Fig. 1), and show a lower greenschist metamorphism degree^11,27^. The Pillahuincó Group is the youngest group, situated on the eastern sector, and the rocks are at diagenetic grade^17,27,39^. Cenozoic deposits unconformably overlie the mentioned units (Fig. 1).

**Figure 3:**
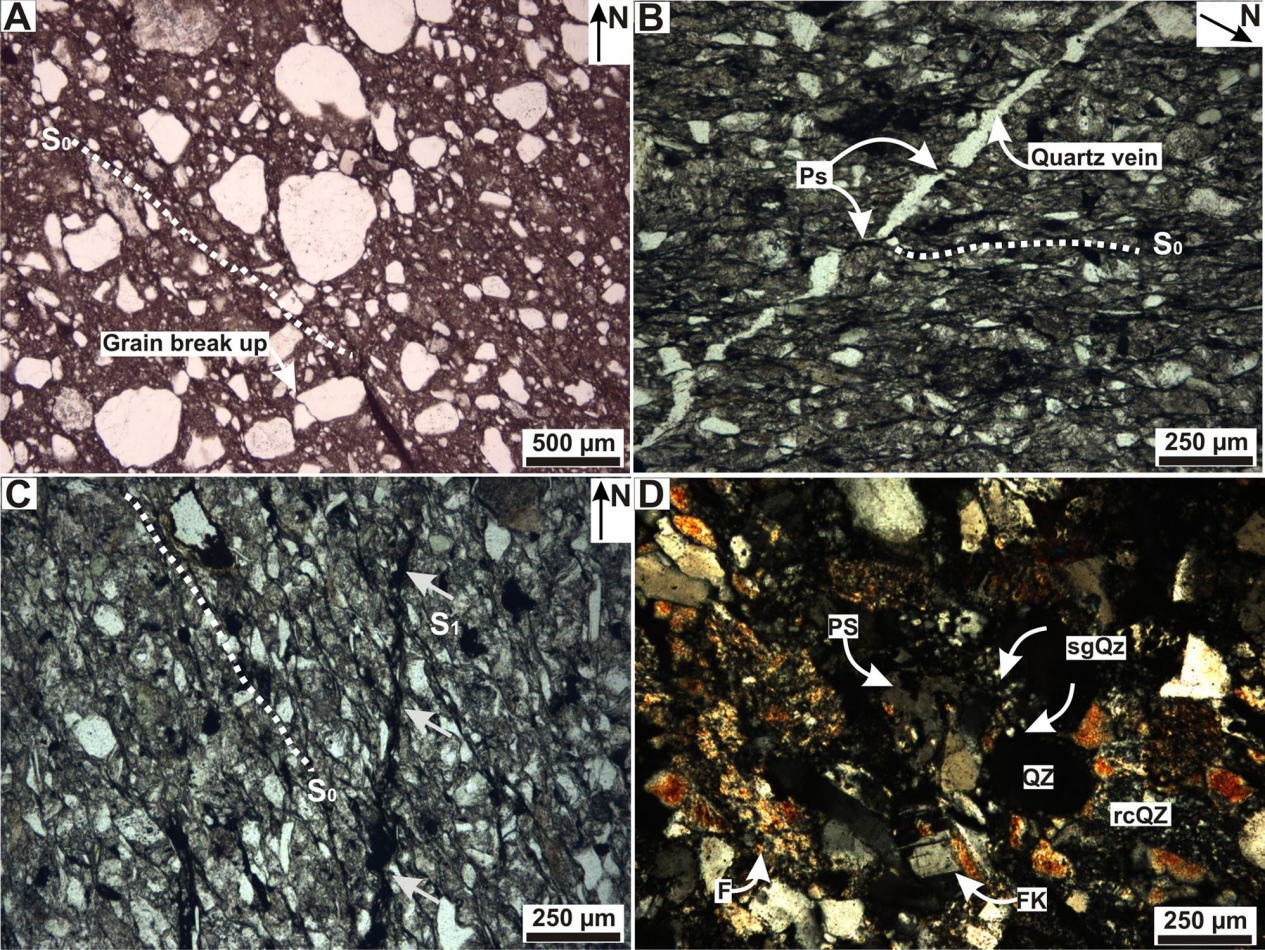
(**A**) Micro-photograph under transmitted light of Sauce Grande Formation. Broad foliation plane (S_0_), braided to parallel shape and penetrative, represent by the phyllosilicates and the opaque minerals (white arrows), with associated grain break up. Micro-photograph of the Piedra Azul Formation. (**B**) Primary foliation (S_0_) represented by phyllosilicates (micas), oxides and veins; quartz veins orientated at 45° with the S_0_ and showing dissolution be pressure (Ps). (**C**) S_0_ and second week foliation plane S_1_ represented by micas and opaque minerals, under transmitted light with parallel nicols. (**D**) Microlithons composed by grains of quartz (Qz) and feldspar (FK), pressure shadows (PS), subgrains in quartz (sgQz) and quartz re-crystallization (rcQz), folia (F) composed by micas, recrystallized quartz, opaque minerals, and epidote, under transmitted light with crossed nicols. The micro-photographs are orientated respect to the north (N).

The Pillahuincó Group^9^ outcrops at the east of the Ventana Ranges (Fig. 1), and is divided in four formations, named from the base to the top: Sauce Grande, Piedra Azul, Bonete and Tunas (Fig. 2). Regional strike of fold axes is NW–SE. At the base of the sequence, folding tends to be cylindrical with shorter wavelength and dipping limbs, while towards the top of the sequence expands and show smoother wavelengths^40^. Cleavage planes trend NW–SE and are nearly vertical, dipping toward the west at the base of the sequence and mostly east on Bonete and Tunas formations (Figs. 2 and 3).

Paleomagnetic studies in the Tunas Formation (top of the Pillahuincó Group) indicate that the magnetizations are syntectonic, with the main grouping of the characteristic remanent magnetization reached at 32% of unfolding at the base of the succession^26^ while at the top of the succession is needed a 90% of unfolding to reach it^25^. This behavior evidences a decrease of the tectonic deformation from the base toward the top of the sequence, consistent with the structural field observations (^40^ and other authors cited in) and AMS results^30,31^.

A Permian volcanism event is preserved the sedimentary record of the Tunas Formation^41,42^ (Fig. 1). Provenance and age data provided by Alessandretti et al.^43^ also indicate the presence of an active pyroclastic activity during Early Permian of Gondwana, which is interpreted as part of distal equivalent of the early episodes of the Choiyoi volcanism, located to the west.

Anisotropy of magnetic susceptibility (AMS) and compaction studies based on petrography in samples of Tunas Formation show a syntectonic deformation, during the Permian, that decreases in intensity toward the foreland located at the east^30,31,32^.

On the Arroyo Atravesado section (Fig. 1B), the Pillahuincó Group is exposed from west to east, base to top, including Sauce Grande, Piedra Azul, Bonete and the transition to the Tunas formation (Fig. 2A). The differences between the formations are based on subtle changes such us the absence of clast in the Piedra Azul respect Sauce Grande or the presence of white motes in the Bonete Formation respect to Piedra Azul. The section is composed by a group of folds with the fold axes trending northwest-southeast. The general dip values of the bedding planes decrease toward the east, where the youngest strata crop out (Fig. 2A,B). No evidence of thrusting in the surveyed sequence is visible^38^. The Sauce Grande Formation^9^ has a thickness of 1100 m and it is composed of diamictite deposits^44–46^, sandstones and a smaller proportion of mudrocks^47^. The palinological content indicates a Pennsylvanian-Cisuralian age^48,49^. The lithic grains are well rounded and have different sizes, between 2 mm and 5 cm, and different composition, as quarzites, granitic rocks, carbonates and mudrocks. The matrix is composed of dark gray siltstone with spaced cleavage. Samples of grains from diamictites and the fine-grain matrix were taken.

Above the Sauce Grande Formation, in transitional contact, is the Piedra Azul Formation, with 300 m thickness^9,50^. It is composed of mudrocks, heterolites and minor dark gray fine sandstones, deposited in a marine environment^51,52^. This unit has a continuous cleavage and intense foliation. The Bonete Formation^9^ lies conformably above the Piedra Azul Formation with a thickness of 400 m. It is composed of fine green arkosic sandstones, with whitish specks, intercalated with dark gray mudrocks^9^. Remains of plants that belong to the Glossopteris Zone indicate an Early Permian age^53^. The sandstones have inverse and cross-bedding stratification and parallel and cross-ripple lamination.

Tunas Formation^9^ which is composed of fine to medium sand grade clastic sedimentites of green, gray, red and yellow colors, with parallel and cross-bedding stratification, and ripple lamination. The sandstones are intercalated with siltstones of red and green colors^52,54,55^, with plants imprints of Glossopteris and Lycopsids, and poor bivalve remains^9,53,56,57^. There are also some tuff levels intercalated in the Tunas Formation sequence^41,58^. Radiometric isotopic dating obtained from tuff outcrops at the upper part of the sequence are 280.8 ± 1.9 Ma^42^ and 284 ± 15 Ma^43^, indicating an Early Permian age. In addition, Arzadún et al.^41^ attained SHRIMP zircon ages of 291.7 ± 2.9 Ma and 295.5 ± 8.0 Ma (Asselian–Sakmarian) from outcrops at the base of the sequence (Ruta 76 locality in Arzadún et al.^30,31,41^) and subsurface. Andreis et al.^55^ measured a thickness of 710 m in the west, while Suero^8^ mentions 2400 m in the southeast sector and Japas^50^ measured 1000 m in outcrop. Zorzano et al.^59^ mention a thickness of 1000 m for this formation and a thickness of more than 960 m in subsurface, not recognizing its base. Lesta and Sylwan^5^ estimated 600 m in Ventana Ranges sector. Significant paleogeographic changes in the basin are only observed inside the Tunas Formation.

## Results

### Petrographic analysis

Three samples of Sauce Grande Formation diamictites, located at the sites JS1, JS3 and JS4 were selected (Fig. 2A). Petrographic analysis shows poor textural and mineralogical maturity, with rounded and sub-rounded grains. Contacts between grains are matrix-supported and the average grain sizes is 500 µm, with maximum of 4 cm and minimum of 100 µm. The grains are mono and polycrystalline quartz, feldspars (microcline and plagioclase), different percent of lithic fragments of metamorphic rocks, mudrocks and minor quantities of volcanic rocks, opaque minerals, carbonate and some mafic minerals. The matrix is silt-size and it is composed of quartz, illite, chlorite, epidote and opaque minerals. Diagenetic illite and epidote are present, superimposed to the grains. The samples belong to the sites JS1 and JS4 classify as lithic diamictites while samples belong to JS3 classify as quarzitic diamictites (Fig. 2C). The samples of Sauce Grande Formation do not have a clear diagenetic foliation plane developed. A spaced foliation orientated 130° N (NW–SE), with a broad development depending on grain size variation of diamictite fabric were observed. Disjunctive rough-shaped cleavage domains are represented by phyllosilicates and opaque minerals, spaced between 1 and 30% and gradational transition between them and microlithons^60^ (Fig. 3A,Table 1). Locally, scarce microfractures are present (grain breakage) aligned to foliation planes (Fig. 3A).

**Table 1:** Outcrop data for each formation of the Arroyo Atravesado locality of 103 specimens and its anisotropy of magnetic susceptibility parameters.

Formation	N_AMS_	B. Plane	Cleavage	Microstructure		K_max_	K_int_	K_min_	e_1_—e_2_—e_3_	K_m_	P_j_ av	T	L	F
				**S**_**0**_	**S**_**1**_									
Sauce Grande	19/19	319/36	145/66	315	340	324/15	266/63	047/22	33.6—34.4—19.8	2.13 × 10^–4^	1.051	0.26	1017	1032
		323/33	153/67											
		321/34	140/75											
		330/31	148/89											
Piedra Azul	29/29	329/25	150/87	334	300	316/04	043/33	232/56	11.4—31.0—30.8	3.19 × 10^–4^	1.077	− 0.099	1040	1034
		331/23	142/75											
		330/30	144/66											
		328/26	152/58											
		333/22	140/66											
		321/53	140/46											
Bonete	43/43	314/40	–	315	339	317/08	220/48	052/41	10.6—42.1—42.0	1.73 × 10^–4^	1.051	− 0.069	1026	1022
		309/16	–											
		312/13	100/90											
		328/10	310/79											
		196/03	340/80											
		135/60	304/45											
		315/87	–											
		320/85	–											
		320/85	126/31											
Tunas	15/15	–	–			318/03	228/13	061/77	14.4—40.0—40.0	8.76 × 10^–5^	1.028	0.173	1011	1014
		193/25	310/80											
		168/22	–											

N_AMS_: number of data (all data were accepted in the analysis). B. Plane: bedding strike (0°–360°) and dip (90° clockwise, from given strike, 0–90°). Cleavage: direction (0°–360°) and dip (90° clockwise, from given strike, 0°-90°). S_0_ and S_1_ foliations measured in thin Sects. (0°-360°). K_*max*_- K_*int-*_ K_*min*_ average orientation of the AMS axes and its confidence ellipses e_1_, e_2_ and e_3_^61^, K_m_ = (K_max_ + K_int_ + K_min_)/3: average susceptibility volume in SI units; P_j_ av: average anisotropy degree (P = K_max_/K_min_: anisotropy degree according to Nagata ^62^; T: average shape parameter of Jelinek^61^; L = K_max_/K_int_: Average lineation; F = K_int_/K_min_: Average foliation (Flinn ^63^). See also figure 2A.

Two samples of Piedra Azul Formation siltstones, located at the sites JP3 and JP6 were selected (Fig. 2A). Petrographic analysis indicates moderate sorting and moderate mineralogical and textural maturity, with angular-shape grains. The contacts between grains are mainly straight to concave–convex and the average size of the grains ranges from 100 to 200 µm, with maximum of 300 µm and minimum of 30 µm. The grains are monocrystalline quartz, potassium feldspar, plagioclase and muscovite. The matrix is composed of quartz and sericite. Diagenetic muscovite and sericite superimposed to the grains and diagenetic epidote were recognized. The samples classify as feldspathic siltstones (Fig. 2C). In the sample of Piedra Azul Formation, from site JP3 (Fig. 2A), a diagenetic foliation related to burial, S_0_, orientated 130° N (NW–SE) is evidenced by oxides, micas and pressure dissolution surfaces. This penetrative plane shows smooth and space cleavage domains, with parallel to anastomosing relationship with microlithons^60^ (Fig. 3B). At 120°N (northwest-southeast), there is 50 µm thickness quartz filled microfracture with diffuse limits. Process of pressure dissolution related to foliation planes resulted in sigmoidal geometry of the vein (Fig. 3B), indicating that the vein formed before the foliation. Another weak foliation plane S_1_ orientated nearly N–S that it is represented by micas and opaque minerals (Fig. 3C). The sample from site JP6 have a smooth, spaced, parallel to slightly anastomosed foliation, with discrete transition between domains and microlithons, orientated at 120° N. Microlithons are composed by grains of quartz and feldspar that depending of its orientation exhibit flattening, pressure shadows, subgrains and pressure dissolution (Fig. 3D, Table 1). Micas, recrystallized quartz, opaque minerals, and epidote compose the folia.

**Figure 4:**
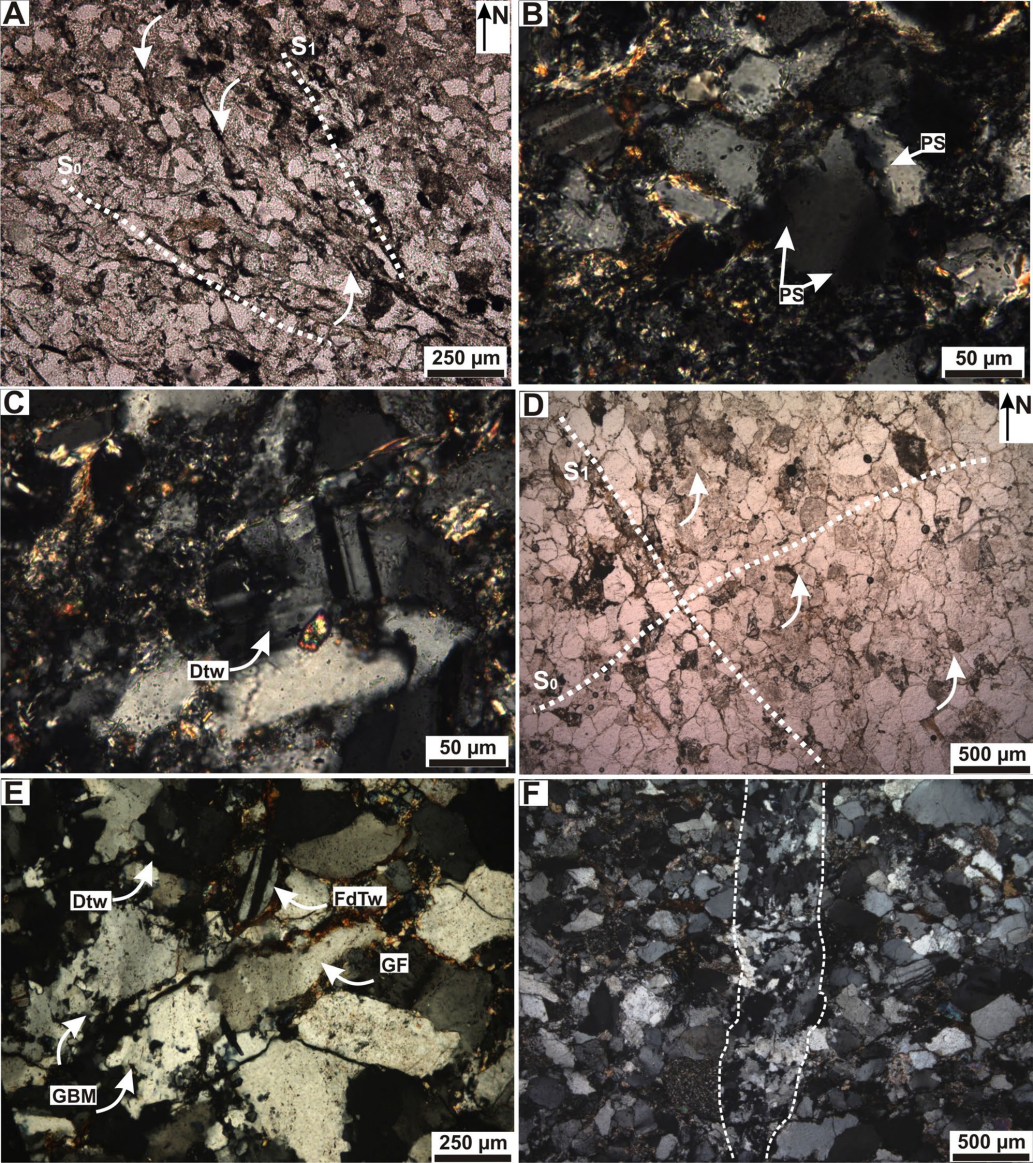
Micro-photograph of the Bonete Formation: (**A**) Primary foliation S_0_ evidenced by flattening of the quartz and feldspar grains in microlithons, micas and opaque minerals in cleavage domains; second foliation S_1_ defined by planes of fluid migration, opaque minerals and micas presence; under transmitted light with parallel nicols. (**B**) Pressure shadows (PS) and subgrains in quartz and feldspar, (**C**) Deformed twins (Dtw). Micro-photograph of the Tunas Formation: (**D**) Primary foliation S_0_ orientated N45°; second foliation S_1_ orientated N20°, defined by planes of fluid migration, micas and opaque; under transmitted light with parallel nicols. (**E**) Grain flattening (GF), deformed feldspar twinning (FdTw) and grain boundary migration (GBM) inside grains under, transmitted light with crossed nicols. (**F**) Vein development with strong grain boundary migration in quartz forming mineral cement phase, under transmitted light with crossed nicols.

Four samples of the Bonete Formation, located at the sites JB3, JB7, JB8 and JB9 were selected (Fig. 2A). Petrographic analysis indicates a good selection with sub-rounded grains. The mineralogical and textural maturity is moderate. The contacts between grains are mainly sutured, with some straight to concave–convex contacts. The average size of the grains is 120 µm, with maximum of 200 µm and minimum of 30 µm. The grains are quartz, potassic feldspar, plagioclase and muscovite, with minor lithic fragments. The matrix is composed of quartz, illite, chlorite and montmorillonite and the cement by opaque minerals, carbonate and diagenetic epidote. Diagenetic chlorite and illite are present. Samples classify as feldspathic sandstones (Fig. 2C). In some sectors there are also wackes, with poor sorting, matrix supported grains and similar composition than sandstones. The sample of Bonete Formation, from the site JB3 (Fig. 2), have a primary foliation S_0_ orientated at 125° N (NW–SE). It is evidenced by flattening of the quartz and feldspar grains in microlithons, mica and opaque minerals that constitutes cleavage domains (Fig. 4A). Foliation observed is spaced, between rough and smooth, and sub-parallel. Transition between the cleavage domains and microlithons is discrete^60^ (Fig. 4A). Pressure shadows and subgrains in quartz grains and feldspar (plagioclase) grains are present (Fig. 4B). There is a second foliation S_1_, 150° N (NW–SE), defined by planes of fluid migration and opaque minerals and micas presence (Fig. 4A, Table 1). The foliation on sample from the site JB7 (S_0_) is evidenced by grains flattening, mica and opaque minerals orientated 130° N (NE–SW) (Table 1). Foliation is spaced, between rough and smooth and sub-parallel. Transition between cleavage domains and microlithons is discrete. Quartz grains present ondulose extinction and subgrains development; feldspars show deformed twins (Fig. 4C). No S_1_ was observed in the site JB7.

Three samples of Tunas Formation sandstones, located at the sites JT1, JT2 and JT3 (Fig. 2A; spanning the meters) were selected. Petrographic analysis indicates moderate to good sorting with sub-rounded grains. The mineralogical and textural maturity is moderate. Contacts between grains are straight to sutured, and the average size of the grains range between 250 and 30 µm, with maximum of 900 µm and minimum of 100 µm. The grains are monocrystalline and polycrystalline quartz, plagioclase, potassium feldspar, lithic fragments of volcanic, granitic and clay rocks, muscovite and epidote. The matrix is composed of sericite, quartz and epidote. There are opaque minerals, silica and epidote as diagenetic cement and there is diagenetic sericite and chlorite. The opaque mineral mainly consists of hematite, that Andreis and Cladera^54^ give a detrital origin. Samples classify as feldspar-lithic and quartzitic sandstone (Fig. 2C).

The samples of Tunas Formation, from sites JT1, JT2 and JT3, show primary foliation parallel to S_0_ 45° N (NE–SW) (Fig. 4D). This is evidenced by flattening of the quartz and feldspar grains in microlithons and mica and opaque minerals in cleavage domains. Foliation observed is spaced, between rough and smooth, and sub-parallel^60^. Transition between the cleavage domains and microlithons is discrete (Fig. 4D). Pressure shadows and subgrains in quartz grains and feldspar (plagioclase) grains with deformation are present (Fig. 4E, Table 1). There is a second foliation S_1_ orientated 130° N (NW–SE), defined by planes of fluid migration, micas presence and opaque minerals (Fig. 4D). Vein development locally show dynamic recrystallization process with strong grain boundary migration in original siliceous cement phase (Fig. 4F).

### Magnetic mineralogy

Diamagnetic and paramagnetic minerals were microscopically recognized in all samples of the Pillahuincó Group, which includes quartz, feldspar, carbonate and phyllosilicates (muscovite, illite, sericite and chlorite). In the samples of the Tunas Formation, the predominant magnetic mineral is hematite (antiferromagnetic mineral), recognized macroscopically and microscopically as detrital grains, cement, nodules and concretions with a detrital and early diagenetic origin^47,54^. The presence of this mineral it is also confirmed by X-ray diffraction analysis^30,31,64^. The average susceptibility measured in this formation is less than 5 × 10^–5^ SI (Table 1; these low values of K_*mean*_ are mainly consistent with contribution of hematite^65^. In the other formations, the average susceptibility measured is between 1.7 × 10^–4^ and 3.2 × 10^–4^ SI, due to the presence of magnetite (Table 1).

**Figure 5:**
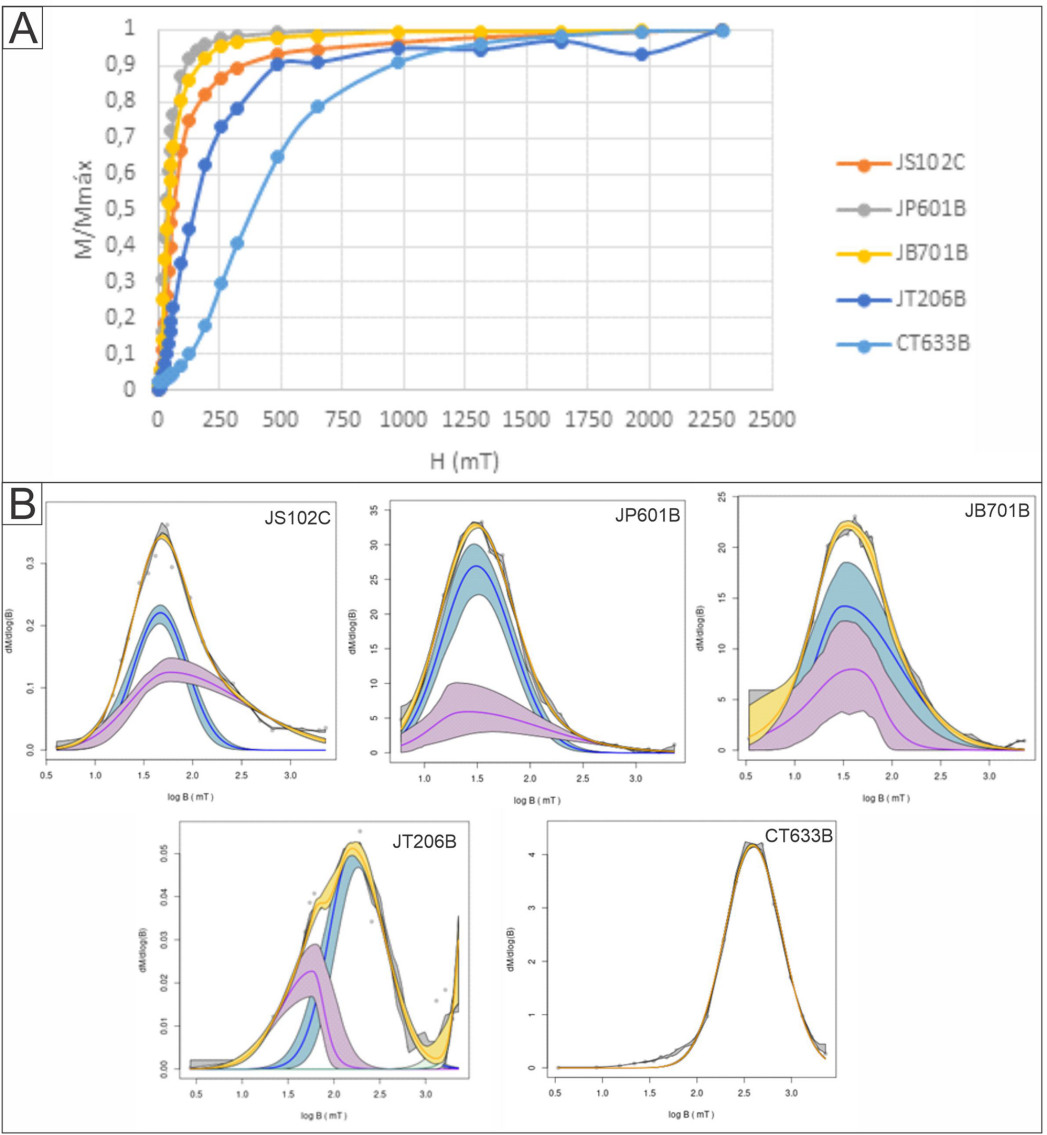
(**A**) Normalized acquisition curves of IRM (Isothermal Remanence Magnetization); H (applied magnetic field), M (susceptibility). (**B**) Model coercivity spectra^66^. Data points are denoted by dots. (Bh = coercivity). The yellow line represents the total coercivity spectra. Blue and purple lines represent de individual contribution of the different coercivity components. Blue and purple shadows represent 95% confidence intervals.

Tomezzoli^25^, obtained natural remanent magnetization intensities between 0.5 and 90 mA m^–1^, in the samples from the Tunas Formation itself, with similar behavior during progressive thermal demagnetization. They were stable during experimental heating, with high magnetic coercivity and unblocking temperatures between 630° and 680 °C, suggesting that the magnetization is carried by hematite. The demagnetization by alternating field was not effective due to the high magnetic coercivity of hematite. Normalized isothermal remanent magnetization (IRM) was performed on five samples: JS102c (Sauce Grande Fm), JP601b (Piedra Azul Fm), JB701b (Bonete Fm), JT206b (Tunas Fm in the Arroyo Atravesado section) and CT633b (Tunas Fm in the San Carlos locality, see Fig. 1) (Fig. 5A). The modeling of coercivity spectra show components with low coercivity on samples JS102c, JP601 and JB701b, and high coercivity on samples JT206b and CT633b (Fig. 5B)^66^. The samples seem to be saturated up to 2 T or less, except those from Tunas Formation (JT206b and CT633b). This behavior is proper of ferromagnetic minerals, probably magnetite, that changes to the Tunas Formation, where the antiferromagnetics minerals (such hematite or non-stoichiometric hematite) begins to manifest. The presence of hematite was also recognized macroscopically and microscopically as detrital grains, cement, nodules and concretions with a detrital and early diagenetic origin^47^, and it was confirmed by X-ray diffraction analysis^30,31,64^.

### AMS data

**Figure 6:**
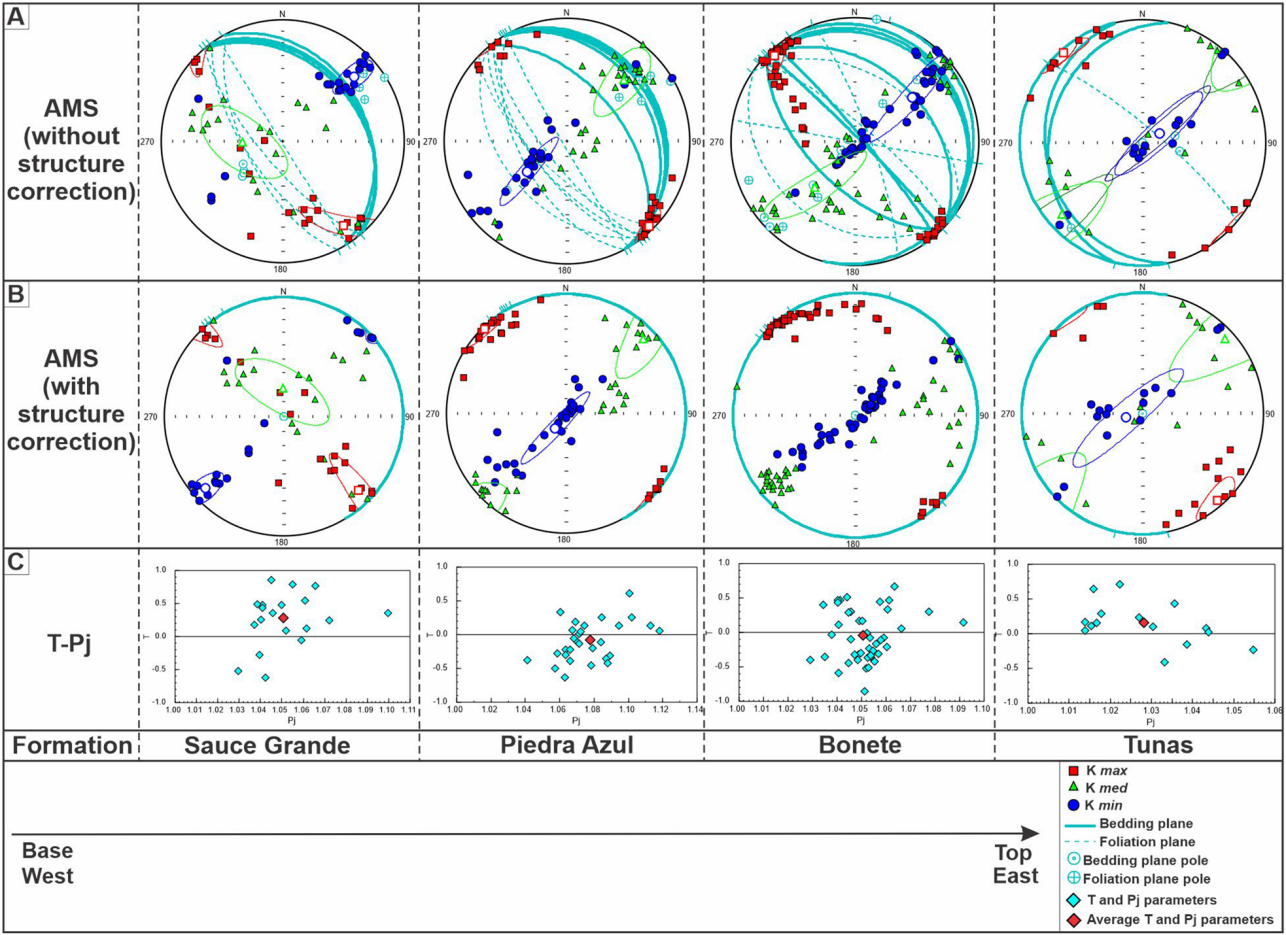
AMS data (Lower hemisphere Schmidt equal area projection) of the different formations of the Pillahuincó Group, on Arroyo Atravesado section (Fig. 1), from de base to the top. (**A**) AMS ellipsoids with its confidence ellipses^61^, bedding planes, foliation and the poles of both planes are showing, (**B**) AMS ellipsoids with structural correction, setting the bedding planes in the horizontal position, with its confidence ellipses^61^, (**C**) ratio between the degree of anisotropy (P_j_) and the shape parameter (T), with the average values in red. Data processed with the Anisoft 5.1.08 software.

**Figure 7:**
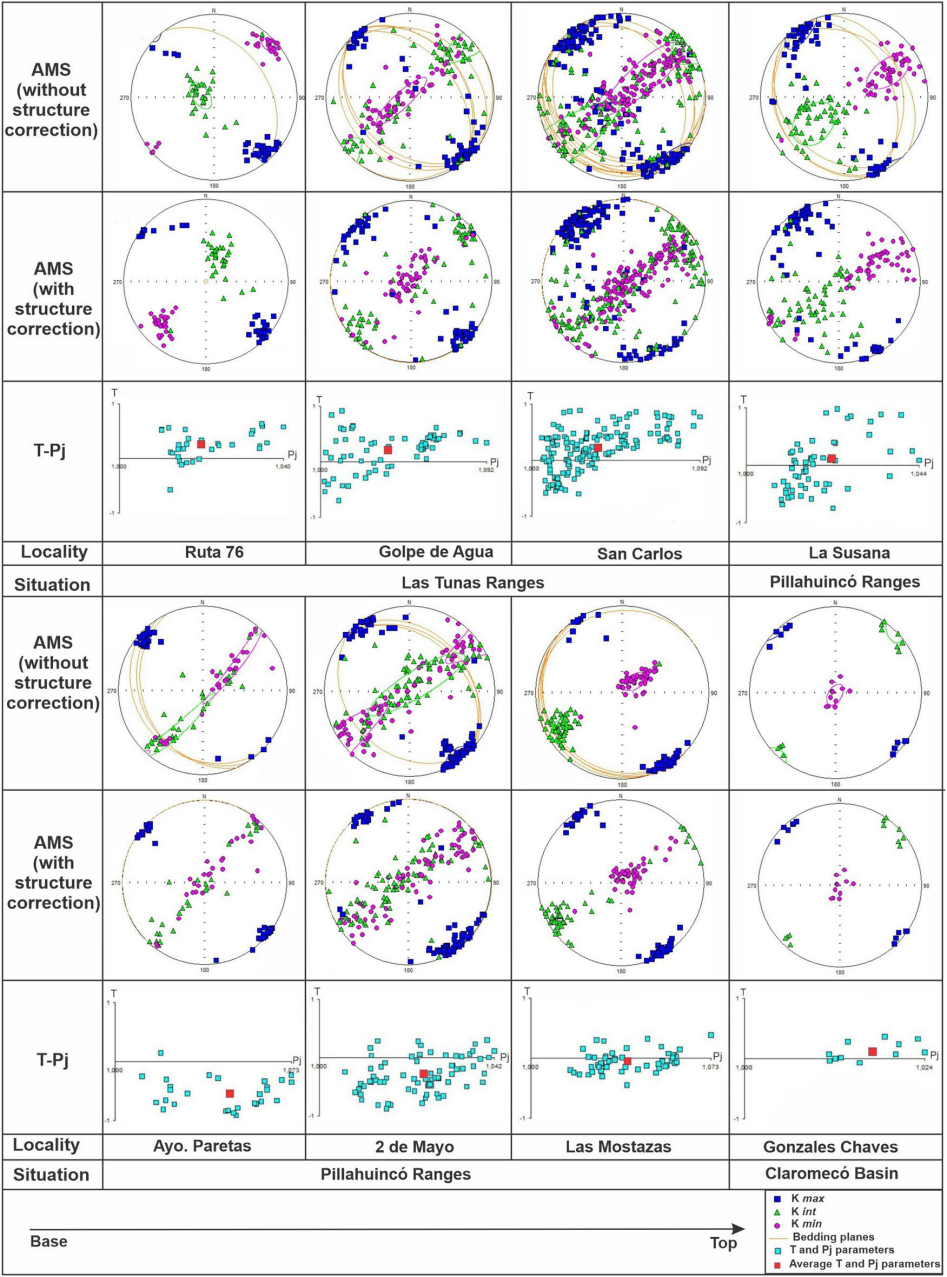
AMS data, with its confidence ellipses^61^, of the different localities of the Tunas Formation situated in Las Tunas Ranges, Pillahuincó Ranges and Claromecó Basin (Fig. 1), from de base to the top: AMS ellipsoids with structural correction, setting the bedding planes in the horizontal position, and ratio between the degree of anisotropy (P_j_) and the shape parameter (T), with the average values in red^30,31^, data processed with the Anisoft 4.2 software).

**Figure 8:**
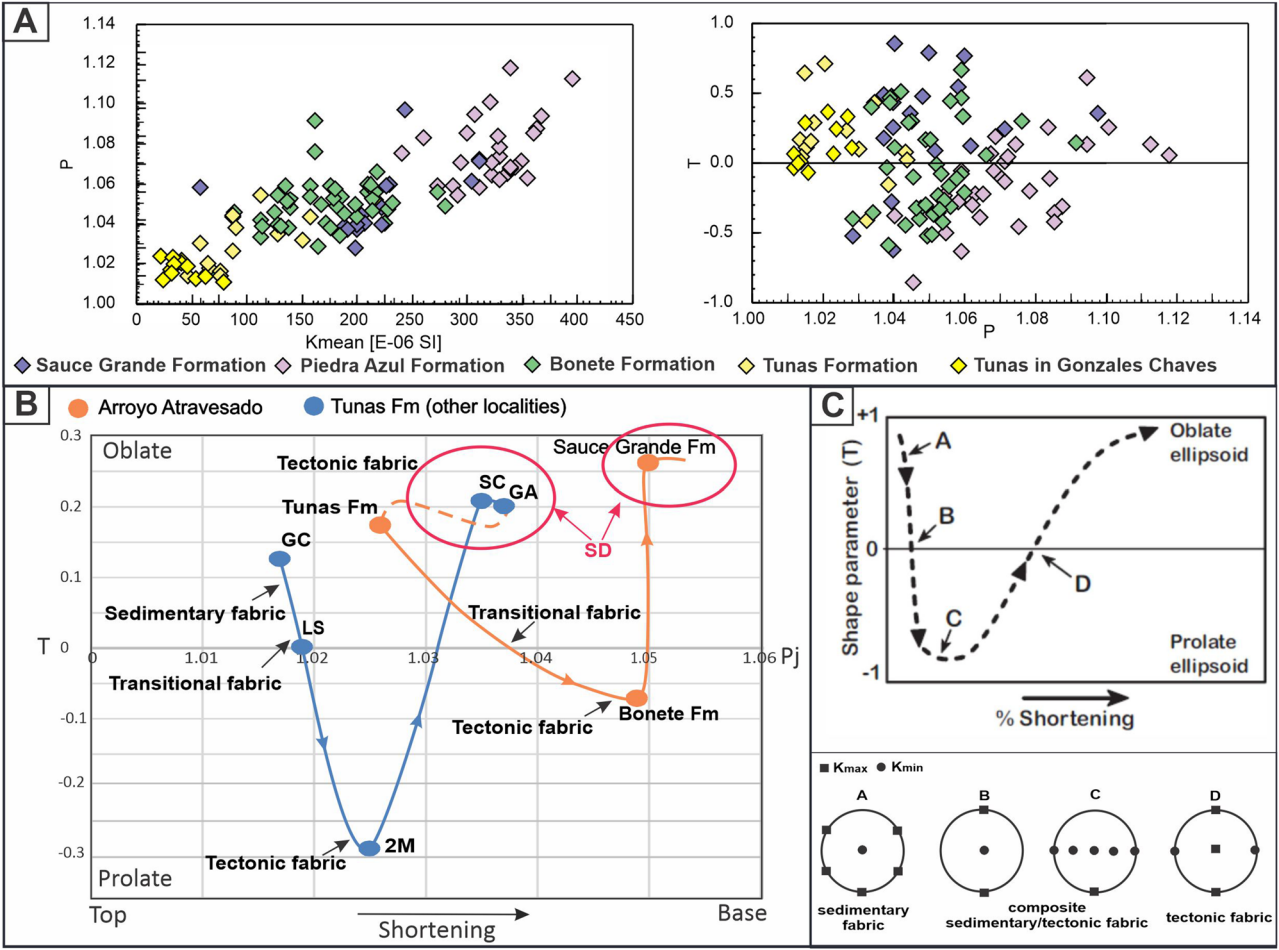
(**A**) Evolution in the ellipsoids according average susceptibility (K_mean_), the shape parameter (T) and the anisotropy degree (P_j_), in the samples of the Arroyo Atravesado log (this work) and in other localities of Tunas Formation^30,31^. Sauce Grande Formation is in the base of the sequence and Gonzales Chaves locality is in the top. (**B**) Simplified curve that shows the evolution of the T and P parameters with the deformation in the Arroyo Atravesado log and some localities of Tunas Formation (GA: Golpe de Agua, SC: San Carlos, 2 M: 2 de Mayo, LS: La Susana, GC: Gonzales Chaves, in^30,31^. The arrow below indicates the direction of the increase of the shortening, as the deformation decrease at the top of the sequence. SD, in red: spasmodic deformation (peaks of greater intensity of deformation). (**C**) Comparison with conceptual models^34–36^. There are changes in the shape of the ellipsoids, from triaxial to prolate and oblate indicating a migration from tectonic to sedimentary fabric in all the cases.

All formations belonging to the Pillahuincó Group, that crop out on the El Atravesado section, show consistent AMS data with well-defined confidence ellipses (Figs. 1 and 2; Table 1). The AMS ellipsoids have maximum axes (K_max_) in northwest-southeast position; this orientation is parallel to the axes of the folds (Fig. 6A,B; Table 1). Sauce Grande Formation presents oblate ellipsoids, with minimum axes (K_min_) grouped in the first quadrant, almost horizontal, suggesting a flattening of the fabric with tectonic control. Moving stratigraphically upwards into Piedra Azul, Bonete and also the base of the Tunas Formation (three sites: JT1, JT2 and JT3 transitional to the Tunas itself described in Arzadun et al. 2016), the ellipsoids tend to change to prolate shapes with a persistent K_max_ in northwest-southeast position, while the K_min_ axes grouped in the first and third quadrant, tends to move toward the vertical (center of the stereographic network), showing a transition to a dominant sedimentary fabric (Fig. 6). However, a few meters above the base of the Tunas Formation, in the Ruta 76 and San Carlos localities (Fig. 1B), the K_min_ axes, oriented NE–SW, lies again close to the horizontal with oblate ellipsoids, suggesting an overlap of tectonic fabric over the sedimentary fabric^30,31^ (Fig. 7). Moving inside the Tunas Formation itself, upwards stratigraphically, in the localities situated to the east, the K_min_ axes moved gradually toward the vertical again, with prolate ellipsoid shapes at first and then with oblate shapes, suggesting again a transitional fabric from tectonic to sedimentary to the top of the sequence^30,31,32^. The dominantly and more clear sedimentary control is in the Gonzales Chaves locality, situated at the Claromecó Basin center (Fig. 1A), where the K_min_ are grouped in the vertical position, perpendicular to bedding planes (Figs. 7 and 8)^30,31,67^.

The anisotropy degree (P_j_) shows a general decrease towards the younger formations and toward the east, with average values from 5% in Sauce Grande Formation to 3% in Tunas Formation. The Piedra Azul Formation has a higher degree of anisotropy than the Sauce Grande Formation, with maximum values of 8%, despite being above in the sequence, probably as a consequence of the finer-grained lithologies presumably more sensitive to the deformation^66–70^ (Fig. 6C; Table 1). Toward the base of the Tunas Formation itself (Ruta 76, San Carlos and Golpe de Agua localities; Arzadún et al.^30,31^; Fig. 7), the degree of anisotropy increases again rising maximum values of 9% to then decrease to the east, toward the top of the sequence, with minimum values of 2% in Gonzales Chaves locality where oblate fabric of sedimentary origin was interpreted^67^ (Figs. 6 and 7).

The shape parameter (T) shows average values larger than zero (T > 0) in the Sauce Grande Formation, indicating oblate shapes of tectonic origin (K_min_ in the horizontal, Fig. 6) that changed to T average values minor than zero (T < 0), toward Piedra Azul and Bonete formations indicating prolate shapes that suggest a transition to sedimentary origin (K_min_ moving to the vertical; Fig. 6 and Table 1). In the base of Tunas Formation itself^30,31^, T values change again to oblate shapes (T > 0) of tectonic origin (K_min_ in the horizontal), that moves to prolate and oblate shapes (transitional to sedimentary fabrics) to the top of the sequence (Fig. 7)^30,31^. Some changes in the AMS patters were observe in the subsurface^32^ confirming that the deformation degree was gradually attenuated upwards in the sequence, to the younger strata and toward the foreland Claromecó basin (Fig. 1).

## Discussion

Along and across the Pillahuincó Group (Upper Paleozoic in the south west of the Gondwana margin) there are evident changes in the outcrops features, micro-tectonic characteristics, types of magnetizations, magnetic mineralogy and AMS signature, from oblate (tectonic) to prolate ellipsoids (transition to sedimentary fabric), towards the top of the sequence located to the eastern and center of the basin^30,31,32^. These changes are also evident in the values of the shape parameter (T), anisotropy degree (P_j_), foliation (F) and lineation (L) depending on location in the stratigraphic sequence and related with the shortening. Even when those parameters are sensitive to mineralogical changes and tectonic strain (^68–71^, between others), should be noted that along the Pillahuincó Group the changes are correlated with the stratigraphic position, independently of the lithology since the lithological differences between the formations are subtle. In the localities situated westward, at the base of the sequence, with major tectonic deformation, the ellipsoids tend to have oblate shapes, changing upwards and eastern positions, to—prolate shapes, and toward the base of the Tunas Formation they have again oblate shapes (Figs. 6, 8A). Previous studies show a similar pattern along the Tunas Formation itself sequence (Fig. 7)^30,31^. In the outcrops, at the base (see Ruta 76 and San Carlos localities in^30,31^), the ellipsoids tend to have oblate-prolate shapes (maximum effort σ_1_ = K_min_ in the horizontal showing tectonic fabric) and upwards they tend to have prolate to oblate shapes (maximum effort σ_1_ = K_min_ in the vertical showing sedimentary fabric). In subsurface, in the foreland Claromecó Basin (Fig. 1), with almost horizontal beds, the spatial distribution of the ellipsoid axis and AMS parameters tend to exhibit equivalent changes from prolate to oblate shapes ellipsoids Febbo et al.^32^ (Figs. 7, 8A). These results and those obtained by Arzadún et al.^30,31^ show a clear pattern that is similar to the theoretical models of Saint-Bezar et al.^34^, Parés and van der Pluijm^35^ and Weil and Yonkee^36^ for weakly to strongly deformed sedimentary rocks in fold and thrust belts. In these models, there are also changes of the AMS response, from oblate shapes in the more tectonically deformed zones to prolate-triaxial and then to oblate shapes. According to Weil and Yonkee^36^, these changes indicate composite sedimentary/tectonic fabrics with layer-parallel shortening (LPS) (Fig. 8B,C).

In all the formations of the Pillahuincó Group the K_max_ axes trend northwest-southeast, parallel to the fold axes and to the primary foliation S_0_, clusters parallel to the intersection of the LPS fabric with bedding, and tend to be constant in all places (Figs. 6, 7). The structure correction in all formations shows a persistence of the K_max_ axis orientation, suggesting a tectonic origin of its behavior with a maximum compressive stress (σ_1_) perpendicular to this axis (Figs. 6, 7). The orientation of the poles of the weak secondary foliation S_1_ coincide with the K_min_, indicating that is related to the shortening. At the base of the sedimentary log, in the westernmost and most deformed localities, the K_min_ axes are almost horizontal, trending southwest-northeast, perpendicular or scatter away from the bedding poles, showing a transition to a tectonic fabric with a maximum compressive stress (σ_1_) in the southwest-northeast direction (Fig. 6), indicating moderate LPS. In contrast, towards the easternmost localities, to the top of the stratigraphic sequence (Bonete Formation and base of Tunas Formation), the K_min_ axes tends to be oriented vertically, showing a transition to a sedimentary fabric and indicating minor LPS (Fig. 6). The microtectonic reveals a primary foliation S_0_ orientated northwest-southeast. The orientation of the S_0_ is coincident with the bedding plane measured in the field, so it is considered as a primary foliation. The secondary foliation S_1_ is penetrative in some samples of the Sauce Grande and Piedra Azul formations, while in the Bonete and Tunas formations is smooth and more spaced, indicating less deformation toward the youngest units. The poles of the S_1_ foliation are coincident with the K_min_ axes of AMS, related with the maximum effort.

Moving towards the Tunas Formation itself, in localities of the base^30,31^, the K_min_ are in a horizontal position again, parallel to the maximum shortening direction with oblate to prolate ellipsoids shape, while at the top of this formation the K_min_ tends to be near vertical positions with prolate to oblate shape (Figs. 7, 8). In the easternmost locality, in Gonzales Chaves^30,31^ and in the subsurface^32^, situated in the center of the Claromecó Basin (Fig. 1), the K_min_ is vertical (Fig. 7). According to Parés^72^ and Weil and Yonkee^36^, this is caused by overburden synchronously with the deformation during the deposition of the sediments (stages D to C of Weil and Yonkee^36^ model Fig. 8C). This is consistent with the decrease of the deformation degree towards the eastern localities.

The changes of the AMS parameters in the sequence of the Arroyo Atravesado locality are in concordance with the characteristics observed microscopically. The foliation in samples of the Sauce Grande and Piedra Azul formations is penetrative, and in Bonete and Tunas formations the foliation is smooth and more spaced (Figs. 3, 4). In addition, these differences are agreed with the different geometry of the folds that is clearly visible in the outcrops along the Pillahuincó Group (Fig. 2). There are also two different types of magnetizations obtained previously in the Tunas Formation^25,26^, from which two different paleomagnetic pole (PPs) positions were calculated: Tunas I PP with 291 Ma (U/Pb ages in^41^, and Tunas II PP with 281 Ma (U/Pb age in López Gamundi et al.^42^ and Arzadún et al.^41^) (Fig. 8).

The differences in the ages of the rocks, the AMS pattern, the presence of syn-tectonic magnetizations, different percentages of unfolding and the changes in the type of foliation demonstrates that the tectonic shortening diminishes towards the top and during a relatively short period, towards the eastern foreland between the Early and Late Permian. Despite this, the K_*max*_ axes remain with constant orientation in the geographic coordinates in all the localities, that means that σ_1_ remains constant from the southwest, at least during that period of time (Figs. 6, 7).

**Figure 9:**
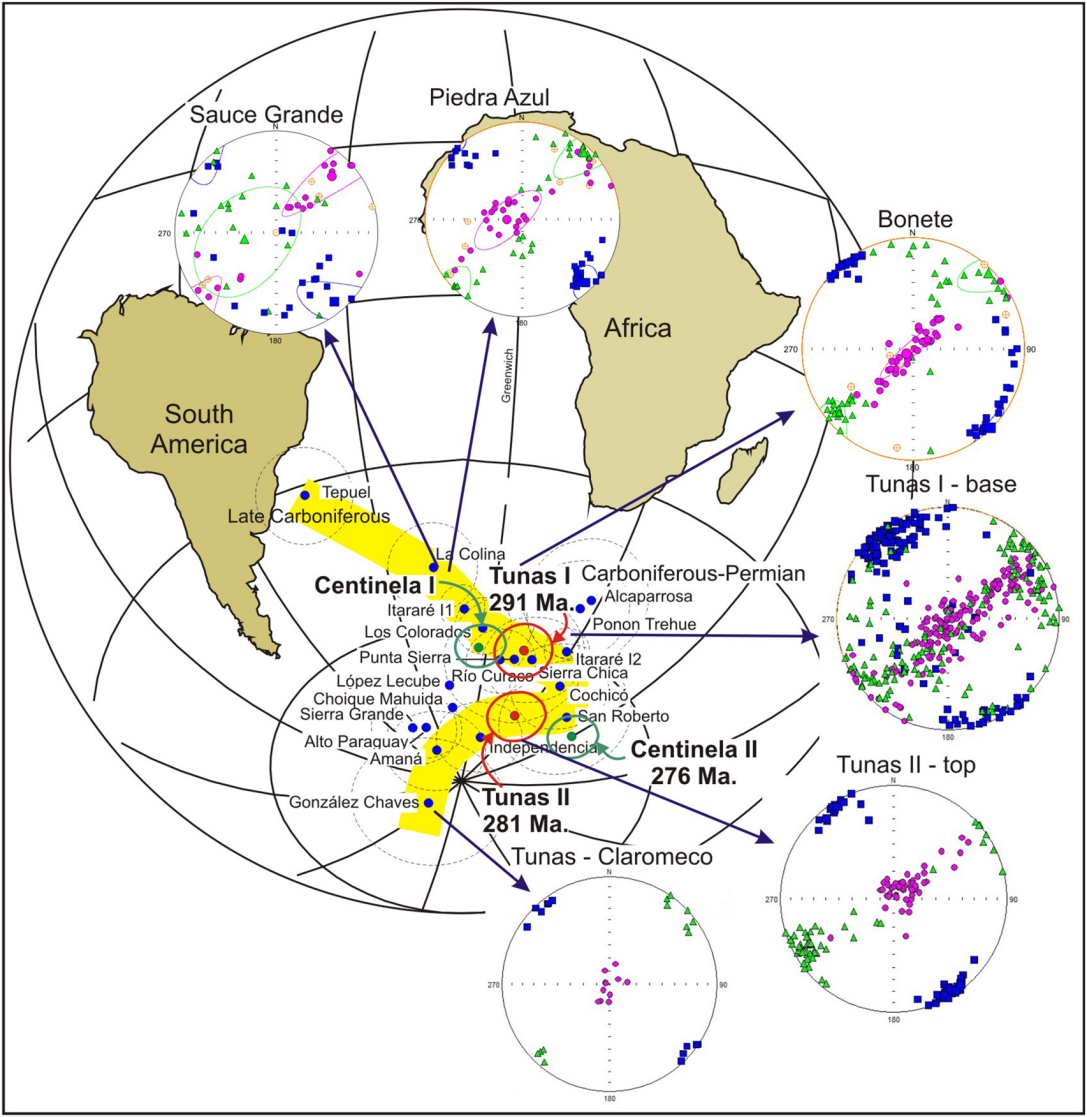
Apparent polar wander path of Gondwana for the Late Paleozoic proposed by Tomezzoli^74^ and Gallo et al.^75^ with the respective ASM patterns (with bedding correction). The abrupt changes in the trajectory of the curve is accompanied by events of greater deformation, evidenced by geological changes in the outcrops, micro-tectonic characteristics, types of magnetizations and different AMS signatures. The shortening direction indicates that the maximum stress along this part of the Gondwana come from the southwest and stay stable during the Lower Carboniferous up to the Permian. These paleogeographic changes associated with that deformation are registered in the cusp that presents the apparent polar wander path of Gondwana during that period^76^. The deformation propagated diachronous eastwards to the foreland, displaying signs first of a decrease and a subsequent pick up of intensity to decays again, suggesting cycles of higher and lower deformation intensity. The first one between the Sauce Grande to Bonete Formations (nearly 300–290 Ma) and the second one insight Tunas Formation (290–276 Ma). The map was made in Gmap (http://www.earthdynamics.org/earthhistory/gmap), and edited in Inkscape (https://inkscape.org/es/).

Similar differences in the AMS patterns were obtained from nearby areas as the Carapacha Basin^33^ (location in the Fig. 1) and in the Sierra Chica locality, belonging to the Choiyoi magmatic Province^73^ (location in Fig. 1), which ages are closer to Tunas Formation (260.8 ± 3.2 Ma to 269.0 ± 3.2 Ma) (Fig. 9). These localities based on the AMS and paleomagnetic results, also show clear tectonic features at the base of the succession, which are attenuated to the top.

According to Tomezzoli^23,25,40,74^, the deformation on the southwestern Gondwana continent margin began during the Late Devonian^77^, and is related with the collision of microplates as Chilenia from the west and Patagonia from the southwest—*CHI-PA* microplate. These collisions give place to the Chañic (Acadic) orogenic phase ocurred during the upper Devonian ^78^. The associated deformation continued until the Late Paleozoic, and is related to the post-collisional San Rafael (Hercinic or Gondwanic) an-orogenic phase in the Late Carboniferous to Middle Permian^78^. The Permian deformation is the consequence of translations movements of the tectonic plates to equatorial positions^76^, which re-organized and adjusted all the plates previously accreted to Gondwana (southern plates) and to Laurentia (northern plates) during the Permian, to configure Pangea during the Triassic^79^. This translation and deformation are reflected in the cusp observed in the apparent polar wander path of South America (Fig. 9)^74^ and Gondwana^75^ during the upper Paleozoic. During the Pillahuinco Group deposition, the geological evidences as macroscopic features of the outcrops, changes in the paleocurrent directions, environmental continentalization, change in the vergence direction to the southwest instead of the northeast, AMS patterns, paleomagnetic poles, minelalogical and microscopic texture chances of the rocks, among other aspects, accompany the Upper Paleozoic palaeogeographical reorganizations of Gondwana (Fig. 9), with two different threshold in the evolution of the basin. The first one between the Sauce Grande and Bonete Formations (nearly 300–290 Ma), and the second one insight the Tunas Formation (290–276 Ma), keeping constant the migration of the orogenic front migration towards the foreland basin during the Carboniferous-Permian.

## Conclusions

As indicated by the AMS ellipsoids, AMS parameters and micro-tectonic analyses, the intensity of the deformation decreases inside the Pillahuincó Group from the Sauce Grande to Bonete and the base of the Tunas Formations (this work), and increases again in the Tunas Formation itself^30,31^.

The AMS results in the Pillahuincó Group (Fig. 8) show K_max_ axes in NW–SE positions, parallel to the axes of the folds and a subhorizontal K_min_ in the base of the sequence, oriented SW–NE indicating a tectonic fabric compatible with the SW-NE regional shortening. To the top of the sequence, the K_min_ move perpendicular to bedding planes indicating the transition to sedimentary fabric. It is interesting to note that above the analyzed column, at the base of the Tunas Formation itself^30,31^, a reactivation of the deformation is found and the K_min_ axis grouped again in the horizontal, indicating a new cycle of greater orogenic activity in the basin during the lower Permian, but always maintaining the SW-NE shortening direction. The K_min_ axes chance from horizontal to vertical from the base to the top of the sequence accompanied with a decrease of the anisotropy degree, indicating an attenuation of the deformation to the younger strata, located at the east of the basin, The provided results represent a further evidence of a migration of the orogenic front towards the foreland basin, acting spasmodically in cycles of higher and lower intensity.

The reactivation of the deformation at the base of Tunas Formation coincides with a latitudinal displacement of Gondwana (from the South) and Laurentia (from the North) continents towards the Equator, between the Lower (nearly 300–290 Ma) and the Upper Permian (290–276 Ma)^75–77^. This is clearly reflected in the cusps that present the apparent polar wander path of South America (Fig. 8) for those times.

## Methods

In order to determine the magnitude and the directions of the deformation stress two different techniques were used: petrographic analysis and anisotropy of magnetic susceptibility (AMS; Tarling and Hrouda^65^). The petrography deals with the interpretation of small-scale features in rocks that yield abundant information on the history and type of deformation^60^. It is possible by this method to describe and measure different characteristics as cleavage and lineation in some minerals, lattice-preferred orientations, deformation mechanisms and kinematic indicators^60,80^. Moreover, the anisotropy of magnetic susceptibility (AMS) is an effective technique used to measure the primary or tectonic petrofabric of the rocks^81^. The method is based on measuring the intensity of magnetization and the direction of magnetic minerals in the rock^82^.

Some selected samples were analyzed by petrography to make microtectonic determinations, using thin polished sections with a Nikon eclipse 50i POL microscope. AMS data were measured in 103 samples, previously sampled for paleomagnetic studies^25,26^. They come from 22 sites occupying different stratigraphic positions of the Pillahuincó Group on the Arroyo Atravesado locality (Fig. 1B, Table 1), 4 sites belonging to the Sauce Grande Formation (19 samples), 6 sites belonging to the Piedra Azul Formation (29 samples), 9 sites belonging to the Bonete Formation (43 samples) and 3 sites belonging to the Tunas Formation (15 samples). In these samples, the principal axes of the AMS ellipsoids (K_max_, K_min_ and K_int_), the shape parameters (T)^61^ and the degree of anisotropy (P) were determined (Fig. 2 and Table 1). This procedure was carried out with a Kappabridge MFK-1A equipment. The obtained data were analyzed by Anisoft 5.1.08. The isothermal remanent magnetization (IRM) was induced using an ASC Model IM-10-30 Impulse Magnetizer successively with a of 3 T coil. The IRM was measured using an AGICO JR-6A Dual Speed Spinner Magnetometer. Modeling of coercivity spectra was performed using a fitting program^66^.

## Data Availability

The data and materials can be requested to the authors, all data are available in its database.

## Abbreviations

AMS: Anisotropy of magnetic susceptibility
Ma: Millions of years
mA m^−^^1^: Magnetization in SI units (milli-ampere per meter)
µm: Micrometers
S_0_: Diagenetic or primary foliation plane
S_1_: Secondary foliation plane
K_max_: Maximum axes of anisotropy of magnetic susceptibility ellipsoid
K_min_: Minimum axes of anisotropy of magnetic susceptibility ellipsoid
T: Shape parameter of anisotropy
P_j_: Anisotropy degree
F: Foliation
σ_1_: Maximum effort of the deformation
LPS: Layer-parallel shortening
PP: Paleomagnetic pole
SD: Spasmodic deformation

## References

[1] Du Toit AL (1927). A Geological Comparison of South America with South Africa.

[2] Pángaro, F., Ramos, V.A., & Pazos, P.J. The Hesperides basin: A continental-scale upper Palaeozoic to Triassic basin in southern Gondwana. *Basin Research*. **28**(5), 685–711 (2015).

[3] Zambrano JJ (1974). Cuencas sedimentarias en el subsuelo de la provincia de Buenos Aires y zonas adyacentes. Revista de la Asociación Geológica Argentina.

[4] Kostadinoff J, Prozzi C (1998). Cuenca de Claromecó. Revista de la Asociación Geológica Argentina.

[5] Lesta, P., & Sylwan, C. Cuenca de Claromecó. VI Congreso de Exploración y Desarrollo de Hidrocarburos. Simposio Frontera Exploratoria de la Argentina. In (Chebli, G.A., *et al* (eds.)) Actas 217–231. (2005).

[6] Pángaro, F., & Ramos, V.A. Paleozoic crustal blocks of onshore and offshore central Argentina: Newpieces of the southwestern Gondwana collage and their role in the accretion of Patagonia and the evolution of Mesozoic south Atlantic sedimentary basins. *Marine and Petroleum Geology*, **37**(1):162–183 (2012).

[7] Ramos VA (1984). Patagonia: un nuevo continente paleozoico a la deriva? 9° Congreso Geológico Argentino (S. C. Bariloche). Actas.

[8] Suero T (1972). Compilación geológica de las Sierras Australes de la provincia de Buenos Aires. Ministerio de Obras Públicas LEMIT División Geología Anales.

[9] Harrington, H.J. Explicación de las Hojas Geológicas 33m y 34m, Sierras de Curamalal y de la Ventana, Provincia de Buenos Aires. Servicio Nacional de Minería y Geología, Boletín 61. (1947).

[10] Rossello EA, Massabie AC (1981). Micro y meso estructuras en las Formaciones Lolén y Sauce Grande y sus implicancias tectónicas en las Sierras Australes de la Provincia de Buenos Aires. Revista de la Asociación Geológica Argentina.

[11] Cobbold PR, Massabie AC, Rossello EA (1987). Hercynian wrenching and thrusting in the Sierras Australes Foldbelt, Argentina. Hercynica.

[12] Japas MS (1995). Evolución estructural de la porción austral del arco de las Sierras Australes de Buenos Aires. Revista de la Asociación Geológica Argentina.

[13] Japas, MS. El Arco Noroccidental de las Sierras Australes de Buenos Aires: Producto de mega kinks extensionales durante el proceso de la deformación? Actas 4° Jornadas Geológicas Bonaerenses 1: 257–263. (1995b).

[14] Japas MS (1999). Revisión de las teorías acerca del origen del arco de las Sierras Australes de Buenos Aires. Revista de la Asociación Geológica Argentina.

[15] Rossello EA, Massabie AC (1992). Caracterización tectónica del kinking mesoscópico de las Sierras Australes de Buenos Aires. Revista de la Asociación Geológica Argentina.

[16] Vizán H, Prezzi CB, Geuna SE, Japas MS, Renda EM, Franzese J, Van Zele MA (2017). Paleotethys slab pull, self-lubricated weak lithospheric zones, poloidal and toroidal plate motions, and Gondwana tectonics. Geosphere.

[17] Von Gosen W, Buggisch W, Krumm S (1991). Metamorphism and deformation mechanisms in the Sierras Australes fold and thrust belt (Buenos Aires Province, Argentina). Tectonophysics.

[18] Chernicoff CJ, Zappettini EO, Santos JOS, McNaughton NJ, Belousova E (2013). Combined U-Pb SHRIMP and Hf isotope study of the Late Paleozoic Yaminué Complex, Rio Negro Province, Argentina: Implications for the origin and evolution of the Patagonia composite terrane. Geosci. Front..

[19] Ramos VA, Chemale F, Naipauer M, Pazos PJ (2013). A provenance study of the Paleozoic Ventania System (Argentina): Transient complex sources from Western and Eastern Gondwana. Gondwana Res..

[20] Álvarez, GT. Cuencas Paleozoicas asociadas a la prolongación norte del sistema de Sierras de Ventania, Provincia de Buenos Aires. PhD thesis. Universidad Nacional del Sur. Argentina. (2004).

[21] Gregori DA, Grecco LE, Llambías EJ (2003). El intrusivo López Lecube: Evidencias de magmatismo alcalino Gondwánico en el sector sudoeste de la provincia de Buenos Aires, Argentina. Revista de la Asociación Geológica Argentina.

[22] Gregori DA, Kostadinoff J, Strazzere L, Raniolo A (2008). Tectonic significance and consequences of the Gondwanide orogeny in northern Patagonia, Argentina. Gondwana Res..

[23] Tomezzoli RN (2012). Chilenia y Patagonia: ¿un mismo continente a la deriva?. Revista de la Asociación Geológica Argentina.

[24] Dimieri, L., Delpino, S., & Turienzo, M. Estructura de las Sierras Australes de Buenos Aires, EN: Geología y Recursos Minerales de la provincia de Buenos Aires, Relatorio del XVI Congreso Geológico Argentino, La Plata, 101–118. (2005).

[25] Tomezzoli RN (2001). Further Palaeomagnetic results from the Sierras Australes fold and thrust belt, Argentina. Geophys. J. Int..

[26] Tomezzoli RN, Vilas JF (1999). Paleomagnetic constraints on age of deformation of the Sierras Australes thrust and fold belt, Argentina. Geophys. J. Int..

[27] Buggisch WE (1987). Stratigraphy and very low grade metamorphism of the Sierras Australes de la Provincia de Buenos Aires (Argentina) and Implications in Gondwana Correlation. Zbl. Geol. Paläont. Teil.

[28] López Gamundi OR, Conaghan PJ, Rossello EA, Cobbold PR (1995). The Tunas Formation (Permian) in the Sierras Australes Foldbelt, east central Argentina: Evidence for syntectonic sedimentation in a foreland basin. J. S. Am. Earth Sci..

[29] Tomezzoli RN, Arzadún G, Cristallini EO (2017). Anisotropía de susceptibilidad magnética y paleomagnetismo en la Formación Lolén de edad Devónica Sierras. Australes de la provincia de Buenos Aires. Revista de la Asociación Geológica Argentina.

[30] Arzadún G, Cisternas ME, Cesaretti NN, Tomezzoli RN (2016). Análisis de materia orgánica en niveles de carbón de la Formación Tunas (Pérmico de Gondwana), Cuenca de Claromecó, Provincia de Buenos Aires, Argentina. Revista de la Asociación Geológica Argentina.

[31] Arzadún G, Tomezzoli RN, Cesaretti NN (2016). Tectonic insight based on anisotropy of magnetic susceptibility and compaction studies in the Sierras Australes thrust and fold belt (southwest Gondwana boundary, Argentina). Tectonics.

[32] Febbo MB, Tomezzoli RN, Calvagno JM, Arzadún G, Gallo L, Cesaretti NN (2021). Anisotropy of magnetic susceptibility analysis in Tunas Formation (Permian) cores, Claromecó Basin, Buenos Aires, Argentina: Its relation with depositional and post-depositional conditions. J. S. Am. Earth Sci..

[33] Tomezzoli RN, Melchor R, MacDonald WD (2006). Tectonic implications of post-folding Permian magnetizations, Carapacha basin, Argentina. Paleomagnetism in Latinoamerica, Special Volume. Earth Planets Space.

[34] Saint-Bezar B, Herbert RL, Aubourg C, Robion P, Swennen R, Frizon D, dede Lamotte DF (2002). Magnetic fabric and petrographic investigation of hematite bearing sandstones within ramprelated folds: Examples from the south atlas front (Morocco). J. Struct. Geol..

[35] Parés JM, van der Pluijm BA (2003). Magnetic fabrics and strain in pencil structures of the Knobs Formation, Valley and Ridge Province, US Appalachians. J. Struct. Geol..

[36] Weil AB, Yonkee A (2009). Anisotropy of magnetic susceptibility in weakly deformed red beds from the Wyoming salient, Sevier thrust belt: Relations to layer-parallel shortening and orogenic curvature. Lithosphere.

[37] Tomezzoli RN, Cristallini EO (1998). Nuevas evidencias sobre la importancia del fallamiento en la estructura de las Sierras Australes de la Provincia de Buenos Aires. Revista de la Asociación Geológica Argentina.

[38] Tomezzoli RN, Cristallini EO (2004). Secciones estructurales de las Sierras Australes de la provincia de Buenos Aires: Repetición de la secuencia estratigráfica a partir de fallas inversas?. Revista de la Asociación Geológica Argentina.

[39] Iñiguez, A.M., & Andreis, R.R. Caracteres sedimentológicos de la Formación Bonete, Sierras Australes de la provincia de Buenos Aires, Reunión Geológica de las Sierras Australes Bonaerenses, Provincia de Buenos Aires, Comisión de Investigaciones Científicas, La Plata 103–120. (1971).

[40] Tomezzoli RN (1999). La Formación Tunas en las Sierras Australes de la Provincia de Buenos Aires. Relaciones entre sedimentación y deformación a través de su estudio paleomagnético. Revista de la Asociación Geológica Argentina.

[41] Arzadún G, Tomezzoli RN, Trindade R, Gallo LC, Cesaretti NN, Calvagno JM (2018). Shrimp zircon geochronology constrains on Permian pyroclastic levels, Claromecó Basin, South West margin of Gondwana, Argentina. J. S. Am. Earth Sci..

[42] López Gamundi OR, Fildani A, Weislogel A, Rossello E (2013). The age of the Tunas Formation in the Sauce Grande basin-Ventana foldbelt (Argentina): Implications for the Permian evolution of the southwestern margin of Gondwana. J. S. Am. Earth Sci..

[43] Alessandretti L, Philipp RP, Chemale F, Brückmann MP, Zvirtes G, Metté V, Ramos VA (2013). Provenance, volcanic record, and tectonic setting of the Paleozoic Ventania Fold Belt and the Claromecó Foreland Basin: Implications on sedimentation and volcanism along the southwestern Gondwana margin. J. S. Am. Earth Sci..

[44] Andreis, RR. Análisis litofacial de la Formación Sauce Grande (Carbónico Superior?), Sierras Australes, provincia de Buenos Aires. Anual Meeting Project 211-IGCP, "Late Paleozoic of South America", San Carlos de Bariloche, Río Negro, Argentina, pp 28–29. (1984).

[45] Coates DA, Amos AJ (1969). Stratigraphy and sedimentation of the Sauce Grande Formation, Sierra de la Ventana, Southern Buenos Aires Province, Argentina. Gondwana Stratigraphy.

[46] Harrington, H.J. Sierras Australes de la Provincia de Buenos Aires. In: *Geología Regional Argentina, Academia Nacional de Ciencias, Córdoba (Reimpresión de Harrington, 1972a)* (Turner, J.C.M., coord.), Vol. 2, 967–983. (1980).

[47] Andreis, R.R., Iñiguez Rodríguez, A.M., Lluch, J.J., & Rodríguez, S. Cuenca paleozoica de Ventania. Sierras Australes de la provincia de Buenos Aires. In Cuencas sedimentarias argentinas. Serie Correlación Geológica (Chebli, G. Spalletti, L. (Eds.)) Vol. 6, 265–298. (1989).

[48] Archangelsky S, Azcuy CL, González CR, Sabattini N, Archangelsky S (1987). Correlación general de biozonas. El Sistema Carbonífero en la República Argentina.

[49] Di Pasquo, M., Martínez, M.A., Freije, H. Primer registro palinológico de la Formación Sauce Grande (Pennsylvaniano-Cisuraliano) en las Sierras Australes, provincia de Buenos Aires, Argentina. *Ameghiniana*. **45**(1):69–81 (2008).

[50] Japas MS (1986). Caracterización geométrico-estructural del Grupo Pillahuincó. I. perfil del arroyo Atravesado. Sierra de Las Tunas, Sierras Australes de Buenos Aires. Academia Nacional de Ciencias Exactas, Físicas y Naturales. Buenos Aires Anales.

[51] Andreis RR, Japas MS (1996). Cuencas de Sauce Grande y Colorado. El sistema Pérmico en la República Argentina y en la República Oriental del Uruguay.

[52] López Gamundi OR (1996). Modas detríticas del Grupo Pillahuincó (Carbonífero tardío-Pérmico), Sierras Australes de la Provincia de Buenos Aires: su significado geotectónico. Revista Asociación Argentina de Sedimentología.

[53] Archangelsky, S., & R. Cúneo. Zonación del Pérmico continental de Argentina sobre la base de sus plantas fósiles, 3° Congreso latinoamericano Paleontológico, México. Memoria, 143–153. (1984).

[54] Andreis RR, Cladera G (1992). Las epiclastitas pérmicas de la Cuenca Sauce Grande (Sierras Australes, Buenos Aires, Argentina). Parte 1: Composición y procedencia de los detritos. Actas 4° Reunión de Sedimentología.

[55] Andreis RR, Lluch JJ, Iñiguez Rodríguez AM (1979). Paleocorrientes y paleoambientes de las Formaciones Bonete y Tunas, Sierras Australes de la Provincia de Buenos Aires, Argentina. Actas 6° Congreso Geológico Argentino.

[56] Furque G (1973). Descripción geológica de la Hoja 34n, Sierra de Pillahuincó, Provincia de Buenos Aires.

[57] Ruiz, L., & Bianco, T. Presencia de restos de Lycopsidas arborescentes en Las Mostazas, Paleozoico Superior de las Sierras Australes, Provincia de Buenos Aires. Primeras Jornadas Geológicas Bonaerenses, Tandil. Comisión de Investigaciones Científicas de la Provincia de Buenos Aires, pp 217. (1985).

[58] Iñiguez, A.M., Andreis, R.R. and Zalba, P.E. Eventos piroclásticos en la Formación Tunas (Pérmico), Sierras Australes, provincia de Buenos Aires, República Argentina. Segundas Jornadas Geológicas Bonaerenses, Bahía Blanca, Actas 1: 383–395. (1988).

[59] Zorzano, A., Di Meglio, M., Zavala, C. , y Arcuri, M.J. La Formación Tunas (Pérmico) en la Cuenca Interserrana. Primera correlación entre campo y subsuelo mediante registros de rayos gamma. XVIII Congreso Geológico Argentino, Actas. Neuquén, 2-6 de Mayo de 2011. (2011).

[60] Passchier, C.W., & Trouw, R.A.J. Microtectonics, Pringer-Verlag, Berlin, pp 366. (2005).

[61] Jelinek K (1981). Characterization of the magnetic Rocks. Tectonophysics.

[62] Nagata, T. 1961. Rock Magnetism. Maruzen Company Ltd., Tokyo, 350 p.

[63] Flinn, D. On folding during three-dimensional progressive deformation. *Quart J. Geol. Sot. London*, **118**, 385–433 (1962).

[64] Arzadún, G., Tomezzoli, R.N., Cisternas, M.E., Cesaretti, N.N., & Fortunatti, N. Análisis diagenético y estructural en la Formación Tunas (Pozo Pang0001-Pérmico de la Cuenca de Claromecó-Sierras Australes, Provincia de Buenos Aires, Argentina). IX Congreso de Exploración y Desarrollo de Hidrocarburos. Mendoza. (2014).

[65] Tarling DH, Hrouda F (1993). The Magnetic Anisotropy of Rocks.

[66] Maxbauer DP, Feinberg JM, Fox DL (2016). MAX UnMix: A web application for unmixing magnetic coercivity distributions. Comput. Geosci..

[67] Arzadún, G., Tomezzoli, R.N., & Cesaretti, N.N. Análisis de anisotropía de susceptibilidad magnética (ASM) y compactación en la Formación Tunas, Sierras Australes de Provincia de Buenos Aires, Argentina. Latinmag Letters, Vol. 3, Volumen Especial, PB18, 1–6. Proceedings Montevideo, Uruguay. (2013).

[68] Borradaile GJ, Henry B (1997). Tectonic applications of magnetic susceptibility and its anisotropy. Earth-Sci. Rev..

[69] Rochette P (1987). Magnetic susceptibility of the rock matrix related to magnetic fabric studies. J. Struct. Geol..

[70] Sagnotti L, Speranza F, Winkler A, Matei M, Funiciello R (1997). Magnetic fabric of clay sediments from the external northern Apennines (Italy). Phys. Earth Planet. Inter..

[71] Hrouda F (1982). Magnetic anisotropy of rocks and its application in geology and geophysics. Geophys. Surv..

[72] Parés JM (2015). Sixty years of anisotropy of magnetic susceptibility in deformed sedimentary rocks. Front. Earth Sci..

[73] Tomezzoli RN, Vizán H, Tickyj H, Woroszylo ME (2013). Revisión de la posición del polo paleomagnético de Sierra Chica en la curva de desplazamiento polar aparente del Gondwana. Latinmag Lett..

[74] Tomezzoli RN (2009). The apparent polar wander path for South America during the Permian-Triassic. Gondwana Res..

[75] Gallo LC, Tomezzoli RN, Cristallini EO (2017). A pure dipole analysis of the Gondwana apparent polar wander path: Paleogeographic implications in the evolution of Pangea. Geochem. Geophys. Geosyst..

[76] Gallo LC, Dalenz Farjat A, Tomezzoli RN, Calvagno JM, Hernández RM (2020). Sedimentary evolution of a Permo-Carboniferous succession in southern Bolivia: Responses to icehouse-greenhouse transition from a probabilistic assessment of paleolatitudes. J. S. Am. Earth Sci..

[77] Tomezzoli RN, Tickyj H, Rapalini AE, Gallo LC, Cristallini EO, Arzadun G, Chemale F (2018). Gondwana’s Apparent Polar Wander Path during the Permian-new insights from South America. Nat.-Sci. Rep..

[78] Azcuy CL, Caminos R, Archangelsky S (1987). Diastrofismo. El sistema Carbonífero en la República Argentina.

[79] Wegener, A. *The origin of Continents and Oceans*. Ed. Methuen, London, pp 212. (1924).

[80] Twiss RJ, Moores EM (1992). Structural Geology.

[81] Graham JW (1954). Magnetic susceptibility, an unexploited element of petrofabric. Geol. Soc. Am. Bull..

[82] Gleizes G, Nédélec A, Bouchez JL, Autran A, Rochette P (1993). Magnetic susceptibility of the Mount Louis-Andorra ilmenite type granite (Pyrenees): A new tool for the petrographic characterization and regional mapping of zoning granite plutons. J. Geophys. Res..

[83] Tomezzoli RN, Saint Pierre T, Valenzuela C (2009). New Palaeomagnetic results from Late Paleozoic volcanic units along the western Gondwana in La Pampa, Argentina. Earth Planets Space.

